# The antimicrobial propeptide hCAP-18 plasma levels in neutropenia of various aetiologies: a prospective study

**DOI:** 10.1038/srep11685

**Published:** 2015-06-29

**Authors:** Ying Ye, Göran Carlsson, Jenny M. T. Karlsson-Sjöberg, Niels Borregaard, Thomas U. Modéer, Mats L. Andersson, Katrin L-A. Pütsep

**Affiliations:** 1 Division of Paediatric Dentistry, Department of Dental Medicine, Karolinska Institutet, Huddinge, Sweden; 2 Childhood Cancer Research Unit, Department of Women’s and Children’s Health, Karolinska University Hospital, Karolinska Institutet, Stockholm, Sweden; 3 Department of Microbiology, Tumour and Cell Biology, Karolinska Institutet, Stockholm, Sweden; 4 The Granulocyte Research Laboratory, Department of Haematology, National University Hospital, University of Copenhagen, Copenhagen, Denmark; 5 School and Hospital of Stomatology, Peking University, Beijing, China

## Abstract

The underlying cause of neutropenia may be difficult to determine due to similar clinical presentation in many neutropenic conditions. The neutrophil protein hCAP-18 (pro-LL-37) is a major component of neutrophil secondary granules and in this prospective study we assessed the use of hCAP-18 levels in blood plasma for differential diagnosis of neutropenic patients (n = 133) of various aetiologies. Plasma levels of hCAP-18 were determined using immunoblot and ELISA. Patients with severe congenital neutropenia (n = 23) presented with the lowest levels of plasma hCAP-18 and differential diagnostic accuracy revealed high sensitivity (100%) and specificity (98.8%) for hCAP-18 ELISA. The correlation coefficient of the hCAP-18 ELISA versus immunoblotting was (R = 0.831) and that of the peptide LL-37 ELISA versus immunoblotting was (R = 0.405) (P < 0.001). Plasma hCAP-18 levels thus displayed high diagnostic value in differential diagnosis of chronic neutropenia. Neutropenic patients with Shwachman-Diamond syndrome, Barth syndrome, Cohen syndrome, acute myeloid leukaemia and specific granule deficiency presented with reduced plasma hCAP-18 levels as well. The blood plasma level of hCAP-18 was thus low in conditions in which the neutrophil antibacterial propeptide hCAP-18 is deficient, *i.e.* severe congenital neutropenia and neutrophil-specific granule deficiency, and in conditions in which bone marrow myelopoiesis is negatively affected.

Neutrophils are innate immune cells of the first line of defence and constitute two thirds of blood leukocytes. Neutrophils are essential in controlling bacterial and fungal infections, and neutrophil deficiency, *neutropenia*, predisposes to severe or fatal infections^1^. Neutropenia may arise in a number of conditions^2^. It may be caused by severe forms of bone-marrow failure or malignant disease^3^,^4^ or emerge as a manifestation of illness secondary to other diseases. It may be an idiopathic condition (idiopathic neutropenia, IN) or a benign childhood condition. Neutropenia may be caused by autoantibodies targeting neutrophil antigens, as in autoimmune neutropenia (AIN), or drug-induced. Different causes of neutropenia may have similar initial clinical presentations, especially in paediatric patients, and establishment of a correct diagnosis is vital. Patient history, detection of autoantibodies, bone-marrow smears and detection of cytogenetic markers of disease all aid in determining a diagnosis^5^,^6^. However, analysis of cytogenetic markers requires specific resources and the detection of autoantibodies often fails due to low serum titers. Since the majority of cases of neutropenia are benign and transient, additional diagnostic markers would be helpful in order to discriminate between the various conditions.

The inherited bone–marrow failure disorder severe congenital neutropenia (SCN) is characterised by circulating neutrophil levels below 0.5 × 10^9^/L, severe recurrent infections and poor prognosis^7^,^8^. Neutropenia in these patients is a consequence of premature apoptosis of neutrophil precursors in the bone-marrow leading to an absence of more mature stages of neutrophil development^9^,^10^. Mutations in *ELANE*, the gene for neutrophil elastase, account for the majority of autosomal dominant SCN while mutations in the gene encoding the HAX-1 protein account for the autosomal recessive forms, also known as Kostmann disease. A few other defined mutations account for a minority of cases^11^. The genetic basis of the disorder SCN remains to be established in approximately 30% of cases^12^,^13^. We have previously demonstrated that the secondary granules in neutrophils of patients with SCN are deficient in hCAP-18 (pro-LL-37, cathelin-LL-37), which is the proprotein of the antimicrobial peptide LL-37^14^. This deficiency, which is due to failed transcription of the *CAMP* gene that encodes for hCAP-18, is a common characteristic of patients with SCN, irrespective of their genetic background^15^. hCAP-18 is readily detectable in blood plasma in healthy individuals^16^ but patients with SCN display severly reduced levels and in a previous pilot study we suggested that reduced plasma hCAP-18 levels could be used to distinguish SCN from AIN and IN^17^. In the present study we assess the use of plasma hCAP-18/LL-37 levels in differential diagnoses of patients with neutropenia of a wide range of aetiologies. Our findings demonstrate that plasma hCAP-18 levels, but not the peptide LL-37 levels, can be used to discriminate the benign conditions of chronic neutropenia from chronic neutropenia caused by severe disease, including severe conditions involving impaired myelopoiesis.

## Results

### Diagnosis outcome

Of the 135 patients included 110 displayed chronic neutropenia as the primary clinical finding and were diagnosed with SCN, AIN, IN, or ethnic neutropenia (EN). (Abbreviations and acronyms used in the present study see Table 1). The clinical characteristics of this group are presented in Table 2. Fifteen of the SCN patients (n = 23) were diagnosed before participating in this study and clinical parameters of these patients have been previously presented^17^. Among these patients, 14/15 received granulocyte colony-stimulating factor (G-CSF) therapy at the time of plasma sampling.

### Plasma hCAP-18 levels and ANC in patients with primary chronic neutropenia

#### Immunoblotting method

**Figure 1:**
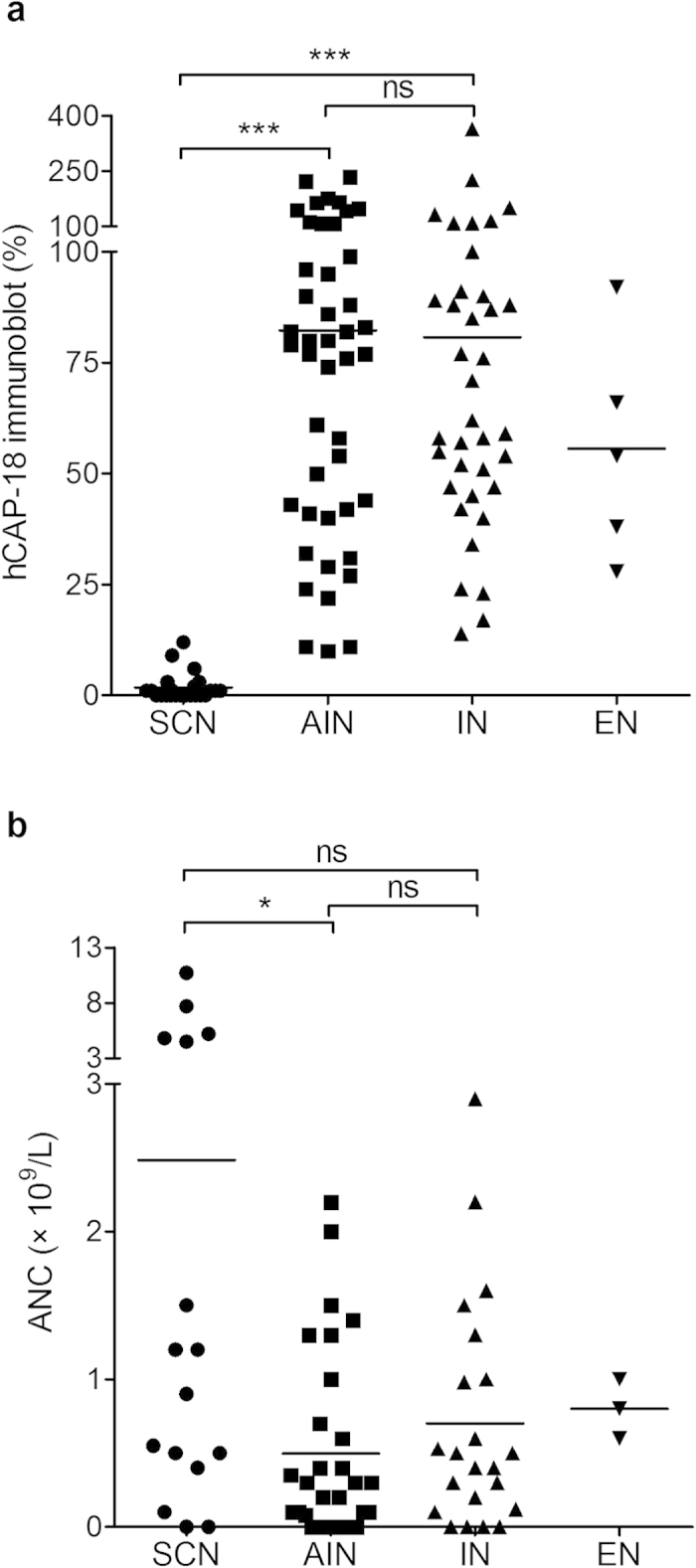
Plasma h-CAP18 levels and absolute neutrophil counts. Plasma hCAP-18 levels (% of standard) assessed by immunoblotting (**a**) and absolute neutrophil count (ANC) (**b**) for patients with chronic neutropenia as the main clinical presentation. SCN, severe congenital neutropenia; AIN, autoimmune neutropenia; IN, idiopathic neutropenia; EN, ethnic neutropenia; ns, no significance. *P < 0.05, ***P < 0.001.

Plasma hCAP-18 levels were significantly lower in patients with SCN (median = 1%, range = 0–12%) compared to those with AIN (median = 79%, range = 10–235%, *P* < 0.001) or IN (median = 62%, range = 14–364%, *P* < 0.001) (Fig. 1a). For individuals with EN who were excluded from the statistical comparison due to the limited number of patients (n = 5), the median value and range of hCAP-18 respectively were 54% and 28–92% of standard (representative Western blots in Supplementary Figure 1). The absolute neutrophil count (ANC) for those patients from whom we received ANC data for the very day of plasma sampling for hCAP-18 is depicted in Fig. 1b. Patients with SCN exhibited higher values (median = 1.05 × 10^9^/l, range = 0–10.7 × 10^9^/l) compared to AIN (median = 0.25 × 10^9^/l, range = 0–2.2 × 10^9^/l, *P* < 0.05), and no significant difference compared to IN (median = 0.45 × 10^9^/l, range = 0–2.9 × 10^9^/l, *P* > 0.05) (Fig. 1b). The wide range of ANC values in the group of SCN patients is most likely a consequence of the patient’s response to and the timing of the G-CSF daily injections. Patients with SCN who were refractory to G-CSF therapy presented with ANC < 0.2 × 10^9^/l. For two patients who received stem cell transplantation, one with SCN and one with neutrophil specific granule deficiency (SGD), we were given the opportunity to analyse plasma samples both before and after the transplantation. Plasma hCAP-18 levels increased from 0% to 68% of reference serum (Supplementary Fig. 1a) and 4% to 20%, respectively, following transplantation.

#### ELISA method

**Figure 2:**
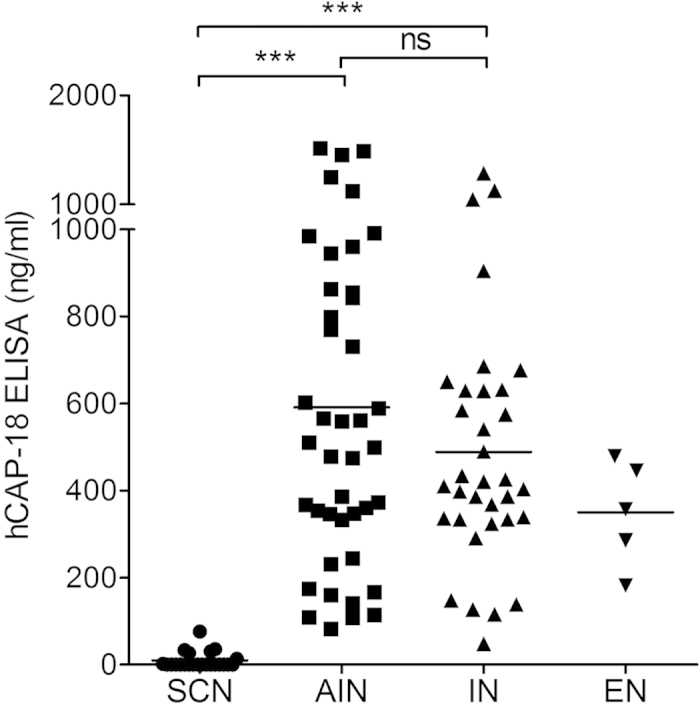
Plasma hCAP-18 levels determined by ELISA. Plasma hCAP-18 levels assessed by hCAP-18 ELISA for patients with chronic neutropenia as the main clinical presentation. SCN, severe congenital neutropenia; AIN, autoimmune neutropenia; IN, idiopathic neutropenia; EN, ethnic neutropenia; ns, no significance. ***P < 0.001.

Using the hCAP-18 ELISA method for plasma hCAP-18 determination, the results mirrored those of the immunoblotting method. The hCAP-18 levels were significantly lower in patients with SCN (median = 0 ng/ml, range = 0–76 ng/ml) compared to those with AIN (median = 505 ng/ml, range = 83–1519 ng/ml, *P* < 0.001) or IN (median = 415 ng/ml, range = 48–1284 ng/ml, *P* < 0.001) (Fig. 2). Individuals with ethnic neutropenia (EN) (n = 5, median = 358 ng/ml, range = 182–479 ng/ml) were again excluded from the statistical comparison due to a too small cohort. Upon comparing hCAP-18 levels between patients with AIN and IN there was no significant difference using either of the assays. The hCAP-18 concentration in the reference standard serum used in immunoblotting was 1016 ng/ml.

### Classification of SCN within primary chronic neutropenia cohort

**Figure 3:**
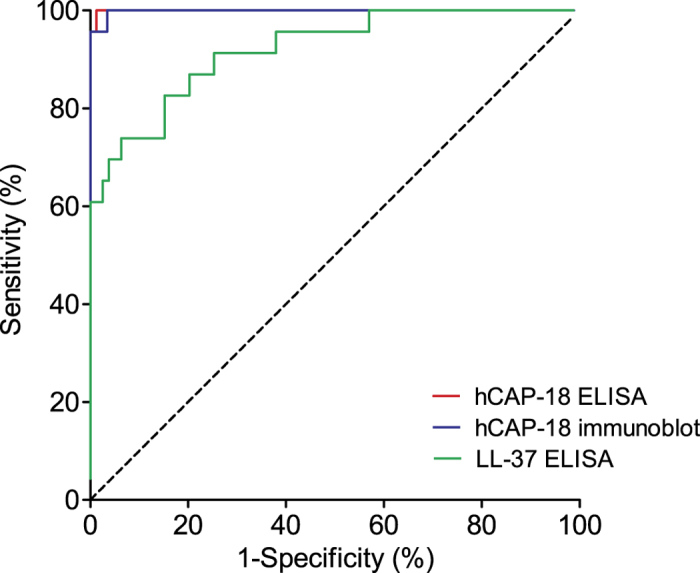
Differential diagnostic accuracy by (ROC) analysis. Receiver operating characteristic (ROC) curves showing performance of plasma hCAP-18 and plasma LL-37 analysis in diagnosis of severe congenital neutropenia for the patient cohort with chronic neutropenia as the main clinical presentation (AIN, IN, SCN, EN).

The receiver operating characteristics (ROC) analysis revealed that the optimum cut-off value for hCAP-18 as SCN diagnosis among the group of patients with chronic neutropenia as the primary clinical presentation (SCN, AIN, IN, and EN) was 88 ng/ml using hCAP-18 ELISA and 14% using immunoblotting (Table 3). For the same diagnostic purpose using the peptide LL-37 ELISA, the optimum cut-off value of LL-37 level was 11 ng/ml (Table 3). In the assessment of differential diagnostic accuracy, hCAP-18 levels using hCAP-18 ELISA had the greatest area under the curve AUC (=0.9995), sensitivity (=100%), specificity (=98.8%), positive predictive value (=95.8%), and negative predictive value (=100%) (Fig. 3 and Table 3).

**Figure 4:**
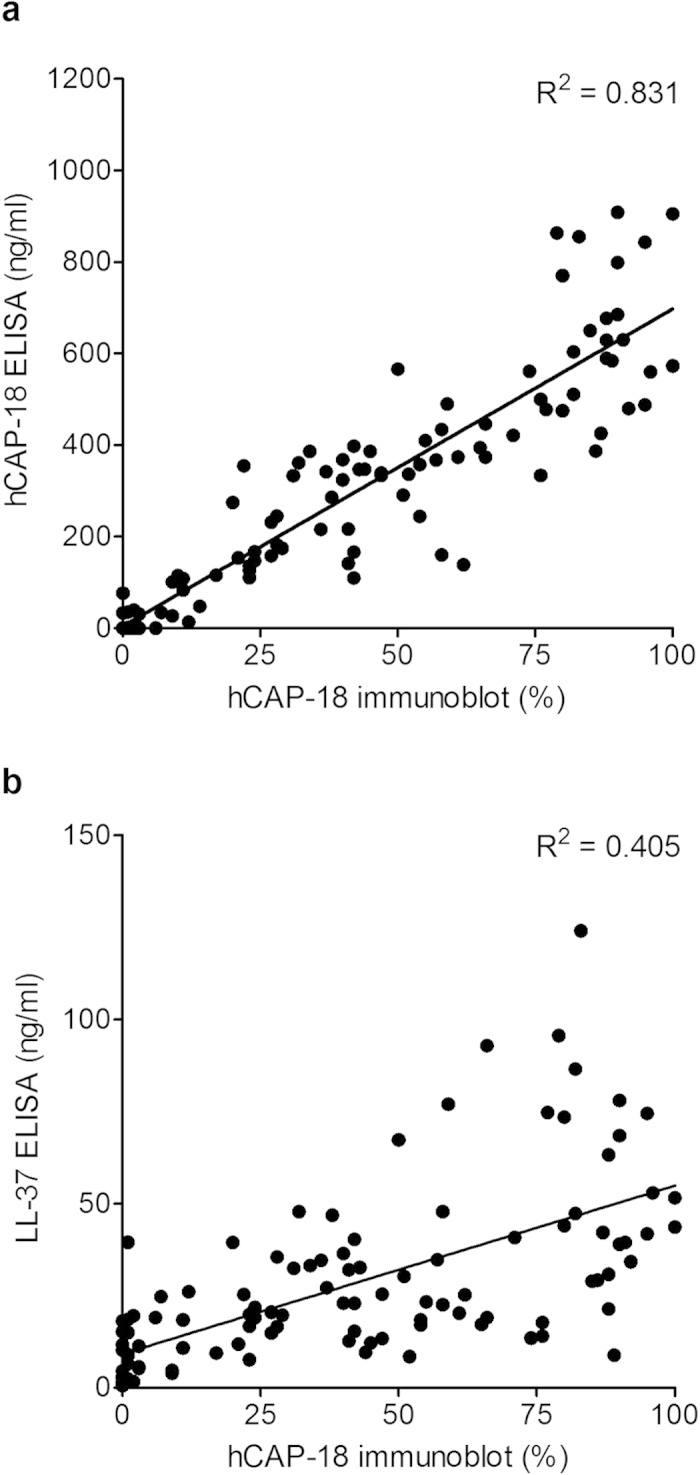
Correlation analysis of plasma hCAP-18 or LL-37 levels versus immunoblotting. The regression lines are between hCAP-18 levels analysed by immunoblot and hCAP-18 ELISA (**a**), and between hCAP-18 levels analysed by immunoblot and LL-37 levels analysed by LL-37 ELISA (**b**).

In the linear regression model correlating hCAP-18 levels analysed by immunoblotting versus hCAP-18-ELISA, the correlation coefficient (R) was 0.831. This is significantly higher than that of correlating hCAP-18 levels analysed by immunoblotting versus the peptide LL-37 ELISA (R = 0.405, *P* < 0.001) (Fig. 4 a,b).

### Plasma-hCAP-18 levels in patients with secondary neutropenia

The diagnoses for individuals with neutropenia secondary to an underlying disease or individuals with inherited bone-marrow failure diseases other than SCN are listed in Table 4. The plasma hCAP-18 levels were reduced in patients with Shwachman-Diamond syndrome, Barth syndrome, Cohen syndrome, SGD, and acute myeloid leukaemia (AML) and similar to those of patients with SCN (Table 4 and Supplementary Fig. 1b–e). In addition, the patient with human herpes virus 6 (HHV6) infection and the patients receiving treatment with thiamazole also presented with reduced plasma hCAP-18 levels. Conversely, the hCAP-18 plasma levels from patients with ataxia telangiectasia, glucose-6-phosphate dehydrogenase deficiency, Graves’ disease or Hyper IgE syndrome, were within the higher range as compared to patients with SCN (Table 4).

## Discussion

In the present prospective study, which encompasses 133 patients with neutropenia lasting more than two months, we assessed the value of using the plasma protein hCAP-18 (pro-LL-37), which is the proprotein of the antibacterial peptide LL-37^18^, as a marker to discriminate neutropenia of various aetiologies. Plasma hCAP-18 mainly arises from differentiating neutrophil precursors in the course of secondary granule protein production in the bone-marrow during myelopoiesis^19^. We demonstrate here that patients with impaired myelopoiesis as well as patients with neutrophil hCAP-18 /LL-37 deficiency present with reduced hCAP-18 plasma levels. Patients with severe congenital neutropenia (SCN) are deficient in neutrophil hCAP-18/LL-37^15^ and this diagnostic group displayed the lowest levels of plasma hCAP-18 among patients with neutropenia as the main clinical presentation. In the diagnostic groups, autoimmune neutropenia (AIN), idiopathic neutropenia (IN) and ethnic neutropenia (EN), the plasma hCAP-18 levels were significantly higher compared to that in SCN. Therapy with recombinant human G-CSF, which leads to rescued myelopoiesis, neutrophil differentiation and elevated ANC values does not correct their hCAP-18/LL-37 deficiency in patients with SCN^14^. Plasma hCAP-18 levels therefore remains low in the patients with SCN who receives G-CSF therapy. Those patients with SCN who are refractory to G-CSF therapy and hence remain severely neutropenic present with low hCAP-18 levels as well^17^. Vitamin D is known to induce production of hCAP-18/LL-37 in hematopoietic cells and we have previously induced the production of hCAP-18/LL-37 by adding vitamin D, *in vitro,* to bone-marrow neutrophil precursor cells from patients with SCN^15^. A concern may arise whether vitamin D supplementation to patients would affect hCAP-18 plasma levels. However, we couldn’t detect any effect on plasma hCAP-18 levels when we attempted supplementation during one month with the hormonal form of vitamin D to a patient with SCN, after which the treatment was discontinued^15^.

Severe congenital neutropenia may be classified as an inherited bone-marrow failure disorder and, interestingly, patients with the inherited bone-marrow failure disorders Barth syndrome, Shwachman-Diamond syndrome and Cohen syndrome similarly presented with low hCAP-18 plasma levels. Barth syndrome is characterised by neutropenia, cardiomyopathy, growth retardation and severe bacterial infections^20^. Barth syndrome is caused by loss-of-function mutations of the tafazzin (*TAZ*) gene and recent data suggests that their neutropenia may be caused by accelerated apoptosis of myeloid precursors^21^. Shwachman-Diamond syndrome is characterised by pancreatic insufficiency and hematopoietic dysfunction^22^. Mutations in the Shwachman-Bodian-Diamond syndrome (*SBDS*) gene, which are associated with Shwachman-Diamond syndrome, lead to impaired myeloid development^23^. Cohen syndrome, finally, is a rare autosomal recessive disorder characterized by facial dysmorphism, microcephaly, truncal obesity, retinopathy and neutropenia^24^. This heterogeneous disorder is caused by mutations in the VPS13B gene, which effect Golgi apparatus function and protein trafficking^25^. Indeed, defective protein trafficking has been suggested as a possible mechanism leading to impaired neutrophil development in congenital neutropenia^26^. In a study of sixteen patients with Cohen syndrome, eight patients displayed an increase of immature neutrophil precursors in the bone-marrow concomitant with fewer more mature stages, indicating defective myelopoiesis in those individuals^27^. Taken together, impaired myelopoiesis is mirrored in low hCAP-18 plasma levels. Moreover, we have recently reported that patients with acute leukaemia presented with reduced levels of hCAP-18 at diagnosis and before initiation of chemotherapy^28^, as did the two acute myeloid leukaemia cases reported here. We consider it likely that the myelopoiesis was disturbed in these patients due to hematopoietic lineage competition and progenitor clonal expansion, reflected as low plasma-hCAP-18 levels. Finally, patients presenting with neutropenia and the diseases ataxia telangiectasia, glucose-6-phosphate dehydrogenase deficiency, Graves’ disease, and Hyper IgE syndrome, displayed hCAP-18 levels in the range of standard sample and no impaired bone-marrow neutrophil differentiation for these diseases have been reported^29^,^30^,^31^,^32^.

For patients with different types of acquired neutropenia we detected reduced plasma hCAP-18 with drugs such as thiamazole, which is known to reduce granulocyte precursors of the bone-marrow^33^. In one case of HHV6 infection the hCAP-18 level was low. It is well-known that several viral infections such as CMV, herpes virus, parvovirus and varicella have a negative impact on the bone-marrow leading to pancytopenia and defective myelopoiesis^34^,^35^. A case of post-bacterial infection neutropenia did not confer reduced hCAP-18 levels, possibly indicating infection-exhausted loss of neutrophils rather than reduced myelopoiesis.

Patients with the neutrophil dysfunction disorder neutrophil-specific granule deficiency (SGD) displayed neutrophil numbers within the range of healthy individuals but they presented with low plasma hCAP-18. This hCAP-18 deficiency parallels that of SCN since patients with SGD have no production of neutrophil secondary granule proteins, including hCAP-18^36^,^37^.

Neutrophils in the blood circulation that are available for ANC determination constitute one pool of bone-marrow produced neutrophils. The other pool is made up of marginated neutrophils, in liver, spleen lungs and other tissues^38^. Neutrophil levels in the circulation may be affected by peripheral destruction or redistributed from the marginal pool^39^,^40^. ANC may thus fail to reflect the bone-marrow myelopoietic activity in a number of clinical situations. Based on our data we suggest that hCAP-18 levels might constitute a stable indicator of myelopoietic activity. In concordance with this suggestion is the observation of increased plasma-hCAP-18 levels following stem cell transplantation of a patient with AML, as reported in a previous study^16^. As may be expected, the plasma levels of hCAP-18 increased following hematopoietic stem cell transplantation of one patient with SCN as well as one patient with SGD in the present study.

Contrary to plasma hCAP-18 levels, detection of the processed peptide LL-37 in plasma tuned out to be less reliable. Using a commercially available ELISA for LL-37 we found lower sensitivity, specificity and predictive value of LL-37 in the differential diagnosis of chronic neutropenia as compared to that of hCAP-18 ELISA or immunoblotting. This is most likely a combined effect of different chemical stability in plasma, due to differences in susceptibility to proteolysis and/or turnover of the proform hCAP-18 versus the peptide LL-3. Both hCAP18 and LL-37 bind to blood plasma lipoproteins but their binding to lipoproteins differ, in that hCAP-18 binds mainly to low-density lipoprotein/ very low density lipoprotein particles in plasma while LL-37 binds to apolipoprotein A1, a major component of high-density lipoproteins^41^,^42^. This difference in binding to apolipoproteins might affect both turnover as well as the antibody recognition of the peptide LL-37 as compared to that of the hCAP-18. Moreover, the antigens recognised by the LL-37 ELISA are within the peptide LL-37 part and could be masked by the cathelin propart of hCAP-18. Our immunoblotting data indicate that little LL-37 is present in the circulation. The LL-37 ELISA will detect hCAP-18 as LL-37 constitutes the C-terminal 37 amino acids of hCAP-18, but the detection is clearly not as robust as with the hCAP-18 ELISA.

In summary, deficiency of plasma hCAP-18 was detected in neutropenic conditions in which the neutrophil antibacterial pro-protein hCAP-18 is deficient, *i.e.* SCN and SGD, and in cases of inherited bone marrow failure syndrome with impaired myelopoiesis. Benign forms of primary chronic neutropenia could thus be distinguished from chronic neutropenia with underlying severe diseases by the analysis of plasma hCAP18 levels. Plasma hCAP-18 levels might also constitute an indicator of myelopoietic activity. We suggest that the use of hCAP-18 as a diagnostic parameter could be developed for clinical use for aiding diagnosis and management of neutropenia and bone marrow failure diseases.

## Materials and Methods

### Participants

This study was designed as a prospective cohort study running over a time-period of ten years. Methods applied were carried out in accordance with the approved guidelines for studies regarding human subjects. All experimental protocols were approved by the Regional Ethics Review Board of the University of Umeå, Sweden (approval Dnr Fek 01-250 and amendment Dnr 2010/146-32M). All subjects have given their informed consent prior to participation in the study.

From January 2003 to March 2013 patients admitted to specialists in paediatric haematology/ oncology were consecutively enrolled and the study encompasses 135 patients. The inclusion criteria were mainly patients under the age of 20 years with neutropenia, *i.e.* absolute neutrophil count (ANC) < 1.5 × 10^9^/l persisting for at least two months. Neutropenia is generally defined as mild when ANC is between 1.0 and 1.5 × 10^9^/L, as moderate between 0.5 and 1.0 × 10^9^/L, and as severe below 0.5 × 10^9^/L. Patients with SCN were included irrespective of age. In addition, two patients without neutropenia but with the neutrophil disorder neutrophil-specific granule deficiency (SGD) were included.

### Diagnosis

Diagnosis of SCN was determined by clinical manifestation, family history, histopathology of bone marrow aspirate and specific gene mutation findings. Autoimmune neutropenia (AIN) was diagnosed by the presence of neutrophil-specific antibodies. Patients without presence of clinical, serological, genetic, or histological evidence of any underlying disease to which neutropenia might be ascribed were diagnosed with idiopathic neutropenia (IN). Ethnic neutropenia (EN) was diagnosed in individuals of African- and Middle East-descent in case there was no evidence of any underlying disease, increased susceptibility to infection or any other adverse effect of the low ANC values. For individuals with neutropenia that was attributed to another underlying disease or condition (Table 4), a diagnosis was established by means of clinical manifestations, family history, laboratory findings, imaging, histological, and if available, genetic analysis.

### Plasma samples

Plasma was collected from EDTA-anticoagulated peripheral blood after centrifugation at 200 g for 10 min at room temperature. Six patients with SCN received hematopoietic stem cell transplantation (HSCT) during the course of this study. The plasma from one of these patients and from one patient with SGD were analysed both pre- and post- HSCT. Plasma analyses were performed by independent researchers blinded from participants’ medical information.

### SDS-PAGE and immunoblot

The plasma was mixed with NuPAGE SDS Sample buffer (Invitrogen, Life Technologies Europe BV, Stockholm, Sweden) containing 10% β-mercaptoethanol and heated at 70 °C before loading. Routinely, 4 μl of plasma or serum was mixed with 76 μl of sample buffer and 10 μl of this mixture was loaded into the gels. Proteins were separated using 1.0 mm 4–12% NuPAGE Bis-Tris Gels (Invitrogen) in NuPAGE MES SDS running buffer (Invitrogen) and blotted onto polyvinylidene difluoride filters (Invitrogen). The filters were blocked in PBS containing 1% Tween 20 supplemented with 2% w/v non-fat dry milk. Immunoblotting was performed using a custom-designed rabbit anti-LL-37 antibody, which also detects hCAP-18^14^, and a horseradish peroxidase-conjugated secondary goat anti-rabbit antibody (Dako AB, Stockholm, Sweden). Bound antibody was detected by chemiluminescence using SuperSignal West Dura (Pierce Biotechnology, Nordic Biolabs AB, Täby, Sweden). Immunoblots were evaluated by densitometry using a Bio-Rad Gel Doc system and Image Lab software (Bio-Rad, Solna, Sweden). Human serum (Sigma-Aldrich, Stockholm, Sweden) was used as the standard for the quantification of hCAP-18. The results were expressed as percentage (%) of the standard.

### HCAP-18 (hCAP-18) ELISA

The hCAP-18 ELISA was conducted with previously generated antibodies directed against recombinant hCAP-18 and recombinant hCAP-18 as the standard^16^. The antibody used is a polyclonal antibody that recognizes both the cathelin propart of hCAP-18 as well as the C-terminal of LL-37.

### LL-37 ELISA

The concentration of plasma LL-37 was assessed using the commercially available human LL-37 ELISA kit (HK321-02, Hycult Biotech, Nordic Biolabs AB, Täby, Sweden) with a detection limit of 0.14 ng/ml and a standard range of 0.14–100 ng/ml. The assay was performed according to the manufacturer’s instructions and plasma was diluted 1:20 before analysis. The antigens recognised by this ELISA are within the peptide LL-37. The entire pro-LL-37 molecule hCAP-18 may also be recognized, provided that the antigen is not masked by the cathelin propart of hCAP-18. The ELISA should thus detect both free LL-37 and variable amounts of hCAP-18.

### Statistical analysis

Differences of independent groups were tested using Kruskal-Wallis non-parametric analysis of variance, followed by multiple comparisons using Dunn’s test. Receiver operating characteristic (ROC) were constructed for hCAP-18 immunoblot, hCAP-18 ELISA, and for the peptide LL-37 ELISA in order to evaluate plasma hCAP-18 or plasma LL-37 levels for diagnosis of SCN, AIN, IN, or EN respectively. The sensitivity, specificity, positive predictive value, negative predictive value, and area under the curve (AUC) were calculated according to the ROC analysis. The optimum cut-off value for diagnosis was estimated by maximising the sum of sensitivity and specificity. The correlation coefficients of the two different ELISAs for plasma hCAP-18 and plasma peptide LL-37 versus immunoblot were calculated using linear regression analysis and coefficients were compared using Fisher’s Z transformation. The statistical software package SPSS version 21 and the GraphPad Prism version 4 were used. All tests were two-tailed with significance assigned at 0.05.

## Additional Information

**How to cite this article**: Ye, Y. *et al.* The antimicrobial propeptide hCAP-18 plasma levels in neutropenia of various aetiologies: a prospective study. *Sci. Rep.* **5**, 11685; doi: 10.1038/srep11685 (2015).

## Supplementary Material

Supplementary Figure 1

## Figures and Tables

**Table 1:** Abbreviations and acronyms.

AIN	Autoimmune neutropenia
AML	Acute myeloid leukaemia
ANC	Absolute blood neutrophil counts
*CAMP*	Gene encoding the hCAP-18 protein
CMV	Cytomegalovirus
*ELANE*	Gene encoding human neutrophil elastase
EN	Ethnic neutropenia
G-CSF	Granulocyte colony-stimulating factor
HAX-1	HS-1-associated protein X-1, (involved in cell survival regulation)
hCAP-18	Human 18-kDa cationic antimicrobial protein, (proprotein of LL-37)
HHV6	Human herpes virus 6
HSCT	Hematopoietic stem cell transplantation
IN	Idiopathic neutropenia
SCN	Severe congenital neutropenia
SGD	Neutrophil-specific granule deficiency

**Table 2:** Characteristics of patients with neutropenia as the main clinical presentation.

Diagnosis	n	Age median (range)	Gender male (%)
Severe congenital neutropenia*	23	10.0 (0.1–27.0)	13 (57)
Autoimmune neutropenia	45	1.1 (0.2–17.5)	19 (42)
Idiopathic neutropenia	37	1.8 (0.1–16.9)	24 (65)
Ethnic neutropenia	5	11.9 (0.7–19.0)	3 (60)

^*^15/23 patients with SCN received G-CSF therapy at sampling.

**Table 3:** Predictive values of plasma hCAP-18 or LL-37 levels* in the diagnosis of SCN.

SCN *versus*AIN, IN and EN	AUC (95% CI)	Sensitivity %	Specificity %	Positive predictive value %	Negative predictive Value %
hCAP-18 ELISA	1.00 (1.00 –1.00)	100	98.8	95.8	100
hCAP-18 IB	0.99 (0.98 –1.00)	100	96.6	88.5	100
LL-37 ELISA	0.95 (0.89 –1.00)	73.9	93.7	77.3	92.5

SCN, severe congenital neutropenia; AIN, autoimmune neutropenia; IN, idiopathic neutropenia; EN, ethnic neutropenia; AUC, area under the curve; CI, confidence interval; IB, immunoblotting.

^*^The diagnostic cut-off values were 14% by immunoblot, 88 ng/ml by hCAP-18 ELISA, and 11 ng/ml by LL-37 ELISA.

**Table 4:** The plasma hCAP-18 levels in patients with neutropenia secondary to an underlying condition or patients with acquired neutropenia, measured by immunoblot.

Diagnosis (n)	hCAP-18 Immunoblot %
Congenital	
Shwachman-Diamond syndrome (1)	0
Barth syndrome (1)	2
Cohen syndrome (1)	20
Ataxia telangiectasia (1)	90
G6PD (1)	42
Graves’ disease (1)	28
Hyper IgE syndrome* (1)	86
Premature infant (2)	23, 66
STAT3, 5a, and 5b deletion (2)	65, 100
Developmental disorders (2)	7, 37
Unspecific immunodeficiency (1)	27
Acquired or therapy-induced	
Acute myeloid leukaemia (2)	3, 23
HHV6 infection (1)	8
Bacterial infection (1)	108
Drug (thiamazole) (2)	9, 21
Drug (low dose cytostatic) (3)	36, 41, 134
Others†	
SGD (2)	1, 4

G6PD, glucose-6-phosphate dehydrogenase deficiency.

^*^The clinical characteristics of this patient has been described previously^32^.

^†^Patients without neutropenia but with neutrophil-specific granule deficiency (SGD).
