# Supplementary material for: The antimicrobial propeptide hCAP-18 plasma levels in neutropenia of various aetiologies: a prospective study
Source: Sci Rep. 2015 Jun 29;5:11685. doi: 10.1038/srep11685 (PMC4484407; doi:10.1038/srep11685)
Supplement: Supplementary Figure 1 [file srep11685-s1.pdf]

The antimicrobial propeptide hCAP-18 plasma levels in neutropenia of various aetiologies: a prospective study

Ying Ye[1,5], Göran Carlsson[2], Jenny M. T. Karlsson-Sjöberg[3], Niels Borregaard[4], Thomas U. Modéer[1], Mats L. Andersson[3], Katrin L-A. Pütsep[3]*

[1]Division of Paediatric Dentistry, Department of Dental Medicine, Karolinska Institutet, Huddinge, Sweden

[2]Childhood Cancer Research Unit, Department of Women's and Children's Health, Karolinska University Hospital, Karolinska Institutet, Stockholm, Sweden

[3]Department of Microbiology, Tumour and Cell Biology, Karolinska Institutet, Stockholm, Sweden

[4]The Granulocyte Research Laboratory, Department of Haematology, National University Hospital,

University of Copenhagen, Copenhagen, Denmark

[5]School and Hospital of Stomatology, Peking University, Beijing, China

*Corresponding author:
Katrin Pütsep, Associate Professor
M1:02, Karolinska University Hospital
171 76, Stockholm
Sweden
Telephone: +46 8-524 871 13
Fax: +46-8-51771220
Email: katrin.putsep@ki.se

Supplementary figure 1. Representative Western blots detecting hCAP-18 in blood plasma

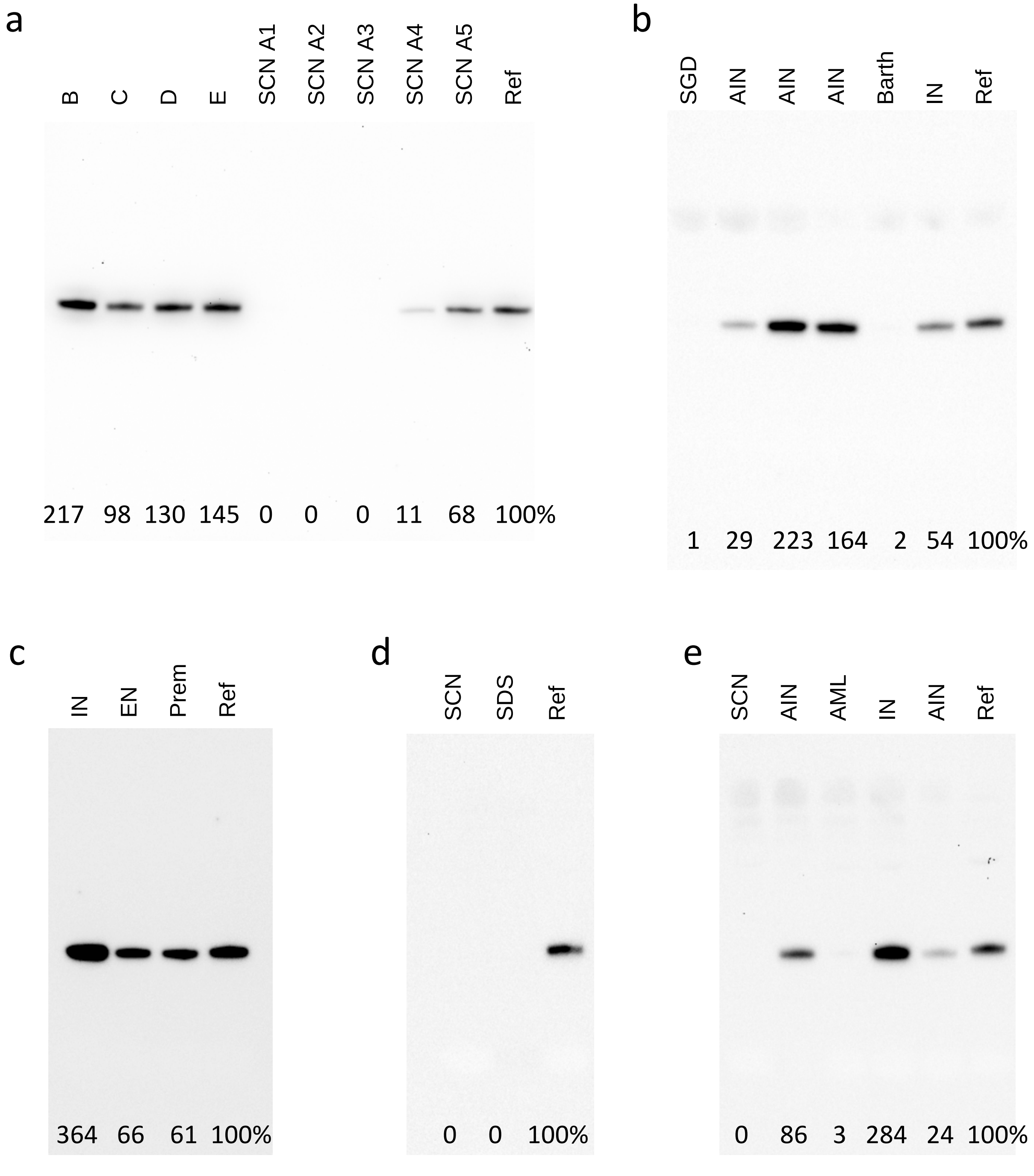

(a) Patient with severe congenital neutropenia (SCN A) and healthy family members B, C, D, E. Lanes SCN A1-A3 are collected from three different occasion with six months apart from the patient SCN A and samples SCN A4 in conjunction with hematopoietic stem cell transplantation and SCN A5 three months later.

(b-e) Patients with chronic neutropenia of various etiologies. Above each lane are the abbreviation for the established diagnosis, AIN; Autoimmune neutropenia, IN; Idiopathic neutropenia, EN; Ethnic neutropenia, SCN; Severe congenital neutropenia, SGD; Neutrophil specific granule deficiency, Barth; Barth syndrome, SDS; Shwachman-Diamond syndrome, AML; Acute myeloid leukemia, Prem; Premature infant. The number at the bottom of each lane corresponds to % of standard reference (Ref) serum sample, determined by Image lab software (BioRad).
